# Mindfulness Meditation Improves Mood, Quality of Life, and Attention in Adults with Attention Deficit Hyperactivity Disorder

**DOI:** 10.1155/2015/962857

**Published:** 2015-06-07

**Authors:** Viviane Freire Bueno, Elisa H. Kozasa, Maria Aparecida da Silva, Tânia Maria Alves, Mario Rodrigues Louzã, Sabine Pompéia

**Affiliations:** ^1^Departamento de Psicobiologia, Universidade Federal de São Paulo, Rua Napoleão de Barros 925, Vila Clementino, 04024002 São Paulo, SP, Brazil; ^2^Hospital Israelita Albert Einstein, São Paulo, Brazil; ^3^Instituto de Psiquiatria, Hospital das Clínicas da Faculdade de Medicina da Universidade de São Paulo, São Paulo, Brazil

## Abstract

*Objective*. Adults with attention deficit hyperactivity disorder (ADHD) display affective problems and impaired attention. Mood in ADHD can be improved by mindful awareness practices (MAP), but results are mixed regarding the enhancement of attentional performance. Here we evaluated MAP-induced changes in quality of life (QoL), mood, and attention in adult ADHD patients and controls using more measures of attention than prior studies. *Methods*. Twenty-one ADHD patients and 8 healthy controls underwent 8 weekly MAP sessions; 22 similar patients and 9 controls did not undergo the intervention. Mood and QoL were assessed using validated questionnaires, and attention was evaluated using the Attentional Network Test (ANT) and the Conners Continuous Performance Test (CPT II), before and after intervention. *Results*. MAP enhanced sustained attention (ANT) and detectability (CPT II) and improved mood and QoL of patients and controls. *Conclusion*. MAP is a complementary intervention that improves affect and attention of adults with ADHD and controls.

## 1. Introduction

The term “mindfulness” has been used to refer to a wide range of phenomena, such as mindfulness as a state, mindfulness as a trait, and mindfulness as a type of training or practice [1], as will be employed here. Interventions based on mindfulness training, such as* mindful awareness practices* (*MAP*), involve intentionally bringing one's attention to one's internal and external experiences in the present moment, and they often involve meditation exercises [2, 3].

Mindfulness-based interventions are considered a type of cognitive training [4] and involve developing strategies that improve attention [5], affective self-regulation [6], and well-being and quality of life [7, 8] in healthy populations [9–11]. Using functional magnetic resonance imaging this type of practice has been shown to improve cognitive control [12] and to establish a stable pattern of deactivation in brain regions related to a mindfulness state [13]. MAP are also beneficial for many clinical conditions, such as anxiety disorders, depression, stress-related physical symptoms, fibromyalgia, chronic pain [14], and attention deficit hyperactivity disorder (ADHD) [4–15]; for a review see [16].

ADHD is characterized by symptoms of inattention, impulsivity, hyperactivity, and affective problems [17–19]. The disorder begins in childhood and can persist until adulthood [17–19] in approximately 50% of patients, and it affects approximately 2–4% of the adult population; see [20].

Regarding affect, ADHD is associated with a lower quality of life [21–23], decreased mood and arousal, and low motivation, all of which can be associated with impaired attentional performance [24, 25] because they relate to cognitive control (conflict resolution, planning, inhibitory control, and error correction) and emotional regulation [26]. In this context, MAP could improve the cognitive/affective processes [6, 27, 28] that are impaired in adults with ADHD [29, 30].

Adults with ADHD show impairment in attentional performance processes considering the influential model of Posner and Petersen [31, 32]. According to these authors, the attention system consists of three functional and anatomically different networks: alerting, the process involved in becoming and staying attentive to one's surroundings, which is closely linked to the concept of sustained attention or vigilance; orienting, or directing attention toward the location or modality of a specific stimulus; and executive attention, which is recruited when there is a conflict among multiple attention cues [31, 32]. These attentional subsystems are classically evaluated using the* Attentional Network Test* (*ANT* [33]). This is a computerized behavioral test that consists of the combination of cued reaction time [34] and the flanker task [35]. Briefly, the ANT involves determining whether arrows presented onscreen are pointing left or right. By measuring how reaction times are influenced by alerting cues, spatial cues, and flanking stimuli (a central arrow was flanked by two arrows pointing either in the same direction or in the opposite direction as the central arrow), the test measures the three attentional networks cited above. This task has determined that executive attention is impaired in adults with ADHD (see [36]), although other studies have shown no impairment [25]. Because a large body of data has shown that performance in this task is clearly related to different brain systems and regions that regulate attention, impairment can indicate physiological changes in brain functioning (see [31, 32]) that cannot be tapped by alterations on subjective measures such as questionnaires.

Another type of computerized attentional test that is widely used to characterize ADHD-induced attentional deficits is* Conner's Continuous Performance Test* (*CPT II* [37]). This test involves a motor response to a series of visual stimuli (letters of the alphabet) and the inhibition of this response to one type of stimulus (the letter x). This test can be used to measure sustained attention (the ability to sustain a consistent focus on continuous activities or stimuli), impulsivity, and selective attention (the ability to focus on relevant stimuli and ignore competing stimuli, a concept related to distractibility). Compared with controls, adults with ADHD make more omission errors on this task [30, 38, 39], present more variability in their mean reaction time [38] and the standard deviation of reaction time [30], are worse at discriminating between target and nontarget stimuli [30], and make more commission errors because of impulsivity or lack of motor inhibition [38–40]. Using measures obtained from the ANT that complement the CPT II, one study showed that adults with ADHD make more omission errors, have lower accuracy and vigilance scores, and present greater variability in responses compared with controls [25]. As with the ANT there are many studies that have associated performance in this test with alterations in specific brain systems. Tana et al. [41], for instance, have shown that performance in this task involves networks consistent with existing models of visual object processing and attentional control. Regarding frontal activation, there was a strong activation in the anterior cingulated cortex, which is particularly important for attentional processing in that it modulates focusing of attention, motor response selection, and error detection. Hence, performance in the ANT and CPT II indicates physiological changes in brain functioning.

The most* accepted* treatment for ADHD according to international guidelines (*NICE Clinical Guideline 072*) is methylphenidate, which improves symptoms. However, other treatments are being sought for a number of reasons: some patients experience side effects that preclude methylphenidate use; others experience only a 30% reduction in symptoms [42]; such stimulants are less effective in adults than in children (see [20]) and some patients are not willing to undergo pharmacological treatment (see [15]). Because many patients undergoing this type of pharmacological treatment still experience functional deficits related to decreases in self-monitoring, attention, and mood, interventions such as MAP that tackle these problems could be used as adjuvant treatments [4, 28].

To our knowledge, however, only two studies [15, 43] have investigated the affective and attentional effects of MAP in adults with ADHD. Zylowska et al. [15] adapted an eight-week group MAP program for this type of patients and showed improvements in ADHD symptoms, executive control (measured using the ANT), and subjective cognitive flexibility and self-regulation. However, that study had no control group, so a practice effect cannot be ruled out. Additionally, the intervention-induced benefits on subjective measures of mood were due to the social interactions of participating in the group sessions. In effect, Mitchell et al. [43], who included a nonintervention group and used the same MAP protocol employed by Zylowska et al. [15], found no evidence of improved attentional performance. They did, however, show that mood benefitted from MAP use. A possible explanation for these conflicting attentional effects is that the type of pharmacological treatment that the patients received was not controlled in these studies, which is important when considering that methylphenidate, the most widely used medication for this disorder, has acute effects on attentional performance [44]. Furthermore, these studies did not include healthy controls, so they could not indicate the measures on which the ADHD patients were impaired in relation to healthy individuals, nor could they establish whether the effects of MAP are differently effective in ADHD patients and in nonclinical populations.

Because meditation has also been shown to improve cognitive efficiency during attentional tasks in the form of less activation in various brain areas [45], it would make sense that practices such as MAP would lead to enhanced attentional performance. Hence, we aimed to investigate the effect of the MAP protocol developed by Zylowska et al. [15] on mood and quality of life (using validated questionnaires), as well as attention (using the ANT and CPT II) in a larger sample of adult ADHD patients of both sexes. We controlled for any acute effects of methylphenidate asking participants on the drug to abstain from their daily dose for 24 h before the study. We also controlled for practice effects by including a control ADHD group that did not participate in the MAP sessions (the nonintervention group). Additionally, to compare the effects of MAP in healthy controls and ADHD patients, two groups of healthy controls were evaluated, one of which participated in MAP and another did not. Based on previous publications [9–11, 15, 43], we hypothesized that MAP would exert positive effects on attentional performance as a proxy for more efficient brain activation during attention tasks [12, 13, 31, 32, 41, 45] in controls and ADHD patients; we also believed that mood would improve in both types of participants. Additionally, we hypothesized that the adults with ADHD would benefit more from the intervention because they have more affective problems and symptoms of inattention and thus would have more room for improvement. We also expected MAP to improve quality of life in patients as a result of better mood and attentional performance, as has been shown for nonclinical samples [7, 8].

## 2. Methods

### 2.1. Participants

All the participants were selected according to the following eligibility criteria: age between 18 and 45 years, more than 11 years of education, normal or corrected vision, nonverbal intelligence quotient (IQ) within normal range [46] (adapted for local use; see Campos [47]), no prior experience with meditation practices, and for whom Portuguese was the native language. Candidates diagnosed with neurological disorders, psychosis, obsessive-compulsive disorder, and Tourette syndrome or who were being treated with psychoactive drugs for reasons other than ADHD were not included in the sample. Participants were also excluded if they scored more than 30 on the Beck Depression Inventory (BDI [48], adapted for local use by Cunha [49]), which indicates severe depression [50] and scored more than the mean plus one standard deviation (see Andrade et al. [51] on the Trait Anxiety Questionnaire of the State-Trait Anxiety Inventory [52], adapted for local use by Biaggio and Natalício [53]).

#### 2.1.1. ADHD Patients

The patients were all diagnosed with ADHD using the Structured Clinical Interview of the DSM-IV and fulfilled the diagnostic criteria of the* DSM-IV-TR* [54]. The diagnosis was made by a psychiatrist who specialized in ADHD. The Adult Self-Report Scale (ASRS; Kessler et al. [55], adapted for local use by Mattos et al. [56]) was used to classify and quantify ADHD symptoms. Some of the patients were recruited by physicians from an adult ADHD diagnosis and treatment program (*Programa Déficit de Atenção e Hiperatividade *(PRODATH) of the Psychiatric Institute of the Universidade de São Paulo (FMUSP)). Other ADHD patients responded to calls for participants in the media.

#### 2.1.2. Healthy Participants

The healthy controls did not fulfill criteria for ADHD but met the other eligibility criteria described above. None of the controls took psychoactive medication during the study.

### 2.2. Procedure

The study was approved by the Ethics Committee of the Universidade Federal de São Paulo and the Universidade de São Paulo (CAAE 20530613.3.3001.0068) and registered at the ClinicalTrials.gov website (Identifier NCT01738334) and at the Registro Brasileiro de Ensaios Clínicos website (Identifier RBR-8dmcnj). All the participants provided informed consent. Information about the included and excluded patients can be found at the beginning of the Results.

Not all of those who were interviewed were willing to participate in MAP, and we were unable to recruit a sufficient number of participants to allow a randomized study. Hence, ours was a quasiexperimental pretest-posttest design with nonequivalent groups in which participants could self-select whether they would or would not participate in the MAP intervention.

Testing took place in the mornings in two one-hour sessions separated by approximately 10 weeks (baseline and endpoint). During this interval, the participants either participated in the eight-week MAP program or underwent no intervention (see details below). Patients treated with methylphenidate took their daily doses in the morning; on testing days they were asked to take their medication only after the experiment. Note that methylphenidate has a relatively short elimination half-life irrespective of its formulation, so that 24 h after their last dose the participants should be free of acute effects of this drug [57].

Affect was measured using validated questionnaires (symptoms of ADHD, mood, and quality of life; see below). The attentional tests administered were the ANT and the CPT II. The tests and questionnaires were administered in a fixed order and were followed by other measures in part of the sample in the preintervention session (a study that investigated ADHD effects on different types of executive functions, Bueno et al. [58]).

Following Zylowska et al.'s [15, 59] protocol, the mindful awareness practices involved weekly two-hour-long group sessions during eight weeks, as well as daily exercises to be performed at home. MAP was conducted on different days for the ADHD patients and the healthy controls.

### 2.3. Mindful Awareness Practices (MAP)

The eight-week group program was adapted from clinical models of mindfulness training [60, 61] by Zylowska et al. [15] and Zylowska [59] to address ADHD-related psychoeducational issues regarding clinical, neurobiological, and etiological symptoms. The material was translated into Portuguese with the permission of the authors, adapted for use in Brazil for both ADHD patients and healthy controls, and was administered by the same highly experienced MAP practitioner. The patients and controls formed separate groups, and the program involved daily exercises to be performed at home (formal meditation and mindfulness in daily living). Each session lasted two and a half hours. Meditation was performed in a seated position, with an emphasis on daily mindfulness. Each session began with a short opening meditation, followed by a discussion about the daily home exercises. After this, new exercises were introduced and practiced by the group in each session, followed by a discussion. Each session ended with a review of the home practice exercises for the following week and a group meditation. All the participants received three CDs to help them with the home meditation practices, which were to be conducted at home for five minutes on weeks one and two, 10 min on weeks three to five, and 15 min on weeks six to eight [15]. The participants kept a diary detailing their meditation at home which allowed us to measure the frequency of the home exercises. At the end of the program, the participants were asked to rate their level of satisfaction with the intervention on a 10-point scale (0 =* totally unsatisfied*; 10 =* totally satisfied*).

### 2.4. Cognitive and Subjective Ratings

#### 2.4.1. Subjective Rating Questionnaires

*Adult ADHD Self-Report Scale (ASRS [55], Adapted for Local Use by Mattos et al. [56])*. This questionnaire consists of 18 items evaluated on a five-point scale ranging from* never *(no symptoms) to* very often* (maximum symptoms). Half of the items evaluate the intensity of usual symptoms of inattention, and the other items evaluate hyperactivity/impulsivity symptoms.

*Beck Depression Inventory (BDI [49], Adapted for Local Use by Cunha [49])*. This is a scale that contains 21 statements regarding symptoms and attitudes related to depression. Each statement is rated on a four-point scale ranging from* neutral* to* maximum severity*. Respondents were asked to rate how they felt in the previous week. 

*State-Trait Anxiety Inventory (STAI-T [52], Adapted for Local Use by Biaggio and Natalício [53])*. This is a self-evaluation scale that contains 20 statements pertaining to anxiety symptoms rated on a four-point scale (1 =* never*; 4 =* always*). 

*Positive and Negative Affect Schedule—Expanded form (PANAS-X [62], Adapted for Local Use by Peluso [63])*. This questionnaire consists of a list of 60 different feelings and emotions. Respondents were asked to rate the extent to which they had these moods during the past week using a five-point scale (1 =* very little or not at all*; 5 =* extremely*). Combinations of these ratings yield two higher-level dimensions (positive affect and negative affect, including 10 feelings each) and 11 lower-order affective levels: fear, sadness, guilt, hostility, shyness, fatigue, surprise, joviality, self-assurance, attentiveness, and serenity. The scores for each dimension were calculated by adding the ratings of all emotions included in each level and dividing the total by the number of emotions in each dimension, so that scores ranged from 1 to 5. 

*Adult ADHD Quality of Life Questionnaire (AAQoL [64], Adapted for Local Use by Mattos et al. [23])*. This scale consists of 29 items rated on a five-point Likert scale (*0 = not at all/never; 5 = extremely/very often; each point receives a score of 25*) that evaluate the level of difficulty in performing activities of daily life grouped into four different areas: life productivity (11 items), psychological health (6 items), life outlook (7 items), and relationships (5 items). Scores for negatively worded items were reversed. Item scores were summed and divided by item count to generate scores for each area and the total score (29 items) and then transformed into 100-point scales. Higher scores indicate better quality of life.

### 2.5. Attentional Tests

#### 2.5.1. Attentional Network Test (ANT [33])

This task was carried out exactly as described in the original work by Fan et al. [33] and took approximately 25 min. Briefly, each trial began with the presentation of a fixation point on the computer screen on which participants were instructed to fix their eyes throughout the trial. After 400 to 1600 ms, an asterisk (cue) could be presented for 100 ms to direct attention to certain areas of the screen that did or did not coincide with the area in which the targets were presented. There were four cue manipulations (see below). Four hundred milliseconds after trials with cues, the target stimulus was presented. This target stimulus was a central arrow presented in a horizontal row including two flanker arrows to either side of the target. These arrows could point either left or right. The participants' task was to indicate the direction in which the central arrow was pointing by using the right or left button of the mouse. These flankers could point in the same direction as the target arrows (congruent condition, which facilitates responses) or in the opposite direction (incongruent condition, which makes the correct response more difficult). The target stimulus remained onscreen for a maximum of 1700 ms or until the participants responded. There was also a control condition that used lines as targets instead of arrows.

Four types of cues conditions influenced task difficulty: no cue, a condition in which only the fixation point was presented and remained on the screen; central cue, in which an asterisk was presented at the same location as the fixation point (this cue involves alerting because it orients the attention to one location); double cue, in which asterisks were presented simultaneously above and below the fixation point (alerting is involved, but the spatial location is broader than in the following condition); and spatial cue, in which the asterisk always occurred in the same spatial location as the target (both alerting and orienting are involved).

The dependent measures were the difference in hit reaction times (RT), that is, when correct responses were given, between the trials in which there were no cue and a double cue (as a measure of alerting), the difference in hit RT between the trials in which there were a central cue and a spatial cue (orienting), and the difference in hit RT between the trials in which there were congruent and incongruent flankers (as a measure of executive control/conflict). Additionally, we analyzed other measures that are typical of the CPT II [37] following Lundervold et al. [25]: (a) reaction time and accuracy: the mean hit RT and the number of hits and omission errors; (b) variability in response: the standard error of the mean hit RT (hit RT SE) and variability SE: the standard deviation of the 3 standard error values calculated for each block; (c) sustained attention/vigilance for interstimulus intervals (ISI) of 400 ms: the slope of the change in RT and in the standard error of the RT between blocks (hit RT block change and hit SE block change, resp.).

Before the participants began the task, they underwent a training session involving 24 trials. The task consisted of three blocks with 96 trials each, separated by a short interval. In each block, the following conditions were randomized: 4 cue conditions × 2 target locations × 2 target directions × 3 flanker conditions × 2 repetitions.

#### 2.5.2. Conner's Continuous Performance Test (CPT II [37])

This task lasts 14 min and consists of 6 blocks in which all letters of the alphabet are presented individually in random order on a computer screen for 250 ms each, with random ISIs of 1, 2, or 4 s. Participants are instructed to press a key whenever a letter is presented, except in the case of the letter x (presented 36 times among the 324 letters presented), for which they should inhibit the motor response. The following measures were recorded: the number of omission and commission errors, the mean hit RT, the variability of standard error (variability of SE), the standard error of the mean hit RT (hit RT SE), detectability (*d*′), response style (*β*), perseverative responses (reaction time less than 100 ms), the slope of the change in RT and in the standard error of RT between blocks (hit RT block change and hit SE block change, resp.), and the slope of change in RT and in the standard error of RT as a function of the ISI (hit RT ISI change and hit SE ISI change, resp.).

### 2.6. Statistical Analyses

The level of significance was *P* ≤ 0.05. We used general linear models (GLM) followed by Tukey's honest significant difference test (HSD) for unequal size samples when factors interacted. The factors and levels will be detailed in the Results. Only significant GLM and* post hoc* effects will be described below. When two or more factors interacted, only the higher-order effects will be described. For measures that showed interactions between intervention and session (there were no interactions of these factors with health status; see below) the magnitude of effects was determined through effect-size calculations (Hedges* g* [65]) following the general rules of thumb to classify effects sizes as small (<0.5), medium (between 0.5 and 0.8), and large (>0.8). These calculations were conducted using change scores (the mean post- minus preintervention scores of participants who underwent MAP and those who did not, divided by the pooled change-score standard deviation), following Mitchell et al. [43]. To assess whether MAP-associated alterations in mood/ADHD symptoms were related to attentional enhancement, we calculated the Pearson product moment correlations between the change scores for attentional measures that benefitted from the MAP and change scores in depression, anxiety, and ADHD symptoms (ASRS).

## 3. Results

**Figure 1:**
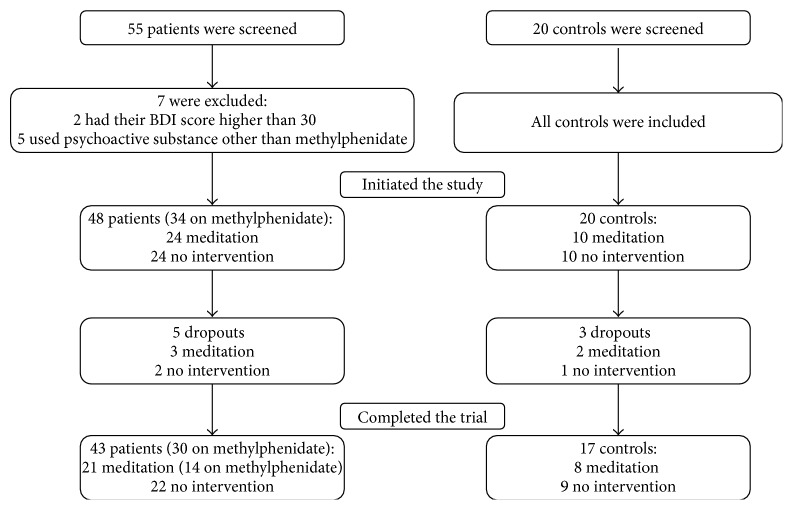
Flowchart of the participants (patients with ADHD and controls).

We screened 55 patients, and seven were excluded (two because their BDI scores were higher than 30 and five because they used psychoactive substances other than methylphenidate). All 20 screened controls were included in the study. Our final sample consisted of 48 patients (34 on methylphenidate) and 20 controls (see the flowchart in Figure 1). Twenty-one patients (11 men) and eight controls (3 men) showed interest in participating in the MAP program. The subjects who did not undergo the MAP intervention between the two testing sessions included 22 ADHD patients (12 men) and nine controls (4 men). Thirteen of the patients had never been medicated for ADHD either by choice or because their condition had not been previously identified; seven of those patients participated in the MAP program. The remainder of the patients used methylphenidate in stable doses for 2 to 60 months (mean ± SD: 16.9 ± 19.8 months), fourteen of whom took part in the MAP.

Two ADHD participants and two healthy controls allocated to the MAP intervention dropped out of the study for personal reasons. Their data were excluded from the analyses. Three control participants (two from the MAP group) did not attend the reevaluation after the intervention period, and their data were excluded. Our final sample consisted of twenty-one ADHD patients (11 men) and eight controls (3 men) who participated in MAP and twenty-two ADHD patients (12 men) and nine controls (4 men) who underwent no intervention. No adverse events associated with MAP were brought to the experimenters' attention. The doses of methylphenidate of the patients taking medication did not change during the study. Fourteen patients (8 men) taking medication were in the intervention group, and sixteen (10 men) were in the nonintervention group.

### 3.1. Comparison of the Demographical Variables and Nonverbal IQ (Preintervention) of Patients and Controls (Table 1)

Demographic information was analyzed using group as factor (ADHD patients who participated in MAP, ADHD patients who did not participate in MAP, healthy controls who participated in MAP, and healthy controls who did not participate in MAP). There were no differences between groups in demographic variables and IQ (all *P* values >0.16). Therefore, differences in attentional performance and subjective measures could not be attributed to these characteristics.

### 3.2. Home Practice and Satisfaction with MAP

See Table 1.

### 3.3. Effects of the MAP Intervention on Subjective Measures (Table 2)

For each dependent measure, GLMs were used which included session (baseline = before the eight-week intervention or nonintervention period; endpoint = after that period) as a within-subject repeated measure factor; health status (ADHD patients or healthy controls) and intervention (MAP or no intervention) were used as between-subjects factors. We focused on the following effects: the interactions of intervention (MAP and no intervention) and session (baseline and endpoint) to determine whether participants willing to participate in MAP differed at baseline from those who were not and whether at the endpoint session measures were improved by MAP; the main effect of session to determine practice effects; the main effects of health status to show the measures in which patients and controls differed; and the interaction of health status, intervention, and session to determine if the MAP was differently effective in patients and controls.

Regarding the questionnaires used in this study, overall, the patients reported more symptoms of ADHD, depression, anxiety, negative mood, and worse quality of life compared with controls. Most of the variables under investigation were sensitive to the MAP intervention, and in the majority of cases this factor interacted with session. With a single exception (subjective inattention on the ASRS), there were no baseline differences between the participants who did and did not participate in MAP. At the endpoint, positive mood and quality of life increased and negative symptoms decreased in the participants who underwent MAP, both in relation to baseline and compared with the participants in the nonintervention condition. There were no interactions between health status, session, and intervention (*P* values >0.08), indicating that there were no significant differences between the ADHD patients and controls. This information will be detailed below.

**Figure 2:**
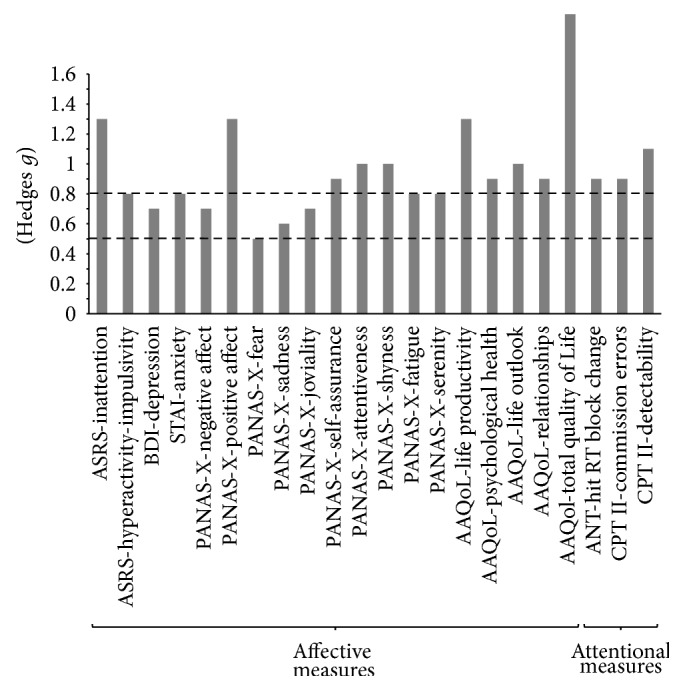
Effect sizes (Hedges* g*) of affective and attentional measures for variables for which there was an interaction of session and intervention factors considering change scores (post- minus preintervention period). Dotted lines indicate medium effect sizes (*g* > 0.5) and large effect sizes (*g* > 0.8). ASRS: Adult Self-Report Scale, BDI: Beck Depression Inventory, STAI: trait anxiety of the State-Trait Anxiety Inventory, PANAS-X: Positive and Negative Affect Schedule—Expanded Form, AAQoL: Adult ADHD Quality of Life Questionnaire, ANT: Attentional Network Task, CPT II: Conner's Continuous Performance Test, and Hit RT: reaction time of correct responses.

The analysis of the ADHD symptoms evaluated by ASRS (Table 2) showed health status effects for inattention (*F*_(1,56)_ = 137.41; *P* < 0.001) and hyperactivity-impulsivity (*F*_(1,56)_ = 32.87; *P* < 0.001), with ADHD patients reporting more symptoms than the controls did. In both cases, there was also an interaction between intervention and session. For inattention (*F*_(1,56)_ = 20.23; *P* < 0.001), the interaction was explained by the fact that, before the intervention, the participants who were willing to undergo MAP had more symptoms than those who were not willing to do so, while the opposite was true after the intervention (*P* values <0.05); there were no session differences among the participants who did not participate in MAP, while symptoms decreased among those who participated in MAP (*P* < 0.01). Inattention was the only measure that indicated a difference at baseline between those who were willing to participate in MAP and those who were not. The effect size on change scores for those who did and did not participate in MAP was large (*g* = −1.3).  Figure 2 shows the effect sizes of all the measures for which there was an interaction between session and intervention.

For hyperactivity-impulsivity scores, there was also an interaction between session and intervention (*F*_(1,56)_ = 7.83; *P* = 0.01). At baseline, there were no significant differences between intervention groups. Additionally, there were no significant differences between the participants who did not undergo MAP. Conversely, at the endpoint, those who participated in MAP reported fewer symptoms compared with their baseline scores and with those who did not participate in MAP (*P* < 0.01). The effect size on the change scores for those who participated in MAP and those who did not was large (*g* = −0.8).

For the depression and anxiety scores obtained from the BDI and STAI-T, respectively (Table 2), we found the same pattern of GLM and* post hoc* effects that we found for hyperactivity-impulsivity symptoms on the ASRS. There was a main effect of health status, indicating that the ADHD patients showed more symptoms on the BDI (*F*_(1,56)_ = 11.83; *P* < 0.001) and STAI-T (*F*_(1,56)_ = 27.26; *P* < 0.001). We also found an interaction between session and intervention for the BDI (*F*_(1,56)_ = 5.79; *P* = 0.02) and STAI-T (*F*_(1,56)_ = 5.59; *P* = 0.02), which indicated no significant difference between conditions at baseline and a MAP-related improvement at endpoint compared with baseline and with the participants who did not undergo intervention (*P* values <0.05), with no significant differences between sessions in the nonintervention condition. Effect sizes for the BDI and STAI-T measure considering the change scores of those who participated in MAP and those did not were medium (*g* = −0.7) and large (*g* = −0.8), respectively.

Regarding the PANAS-X (Table 2), there was a main effect of health status for most variables, which indicated worse mood in the ADHD patients (negativeaffect: *F*_(1,56)_ = 9.54, *P* < 0.001; positive affect: *F*_(1,56)_ = 4.88, *P* = 0.03; sadness: *F*_(1,56)_ = 4.95, *P* = 0.03; joviality: *F*_(1,56)_ = 10.08, *P* < 0.001; self-assurance: *F*_(1,56)_ = 4.72, *P* = 0.03; attentiveness: *F*_(1,56)_ = 15.80, *P* < 0.001; fatigue: *F*_(1,56)_ = 4.41, *P* = 0.04; and serenity: *F*_(1,56)_ = 14.05, *P* < 0.001). For these same variables, plus shyness and fear, we also found interactions between session and intervention (negative affect: *F*_(1,56)_ = 7.49, *P* = 0.01;* g* = −0.7; positive affect: *F*_(1,56)_ = 18.13, *P* < 0.001; *g* = 1.3; sadness: *F*_(1,56)_ = 7.92, *P* = 0.01; *g* = −0.6; joviality: *F*_(1,56)_ = 7.82, *P* = 0.01; *g* = 0.7; self-assurance: *F*_(1,56)_ = 9.05, *P* < 0.001; *g* = 0.9; attentiveness: *F*_(1,56)_ = 6.50, *P* = 0.01; *g* = 1.0; fatigue: *F*_(1,56)_ = 4.80, *P* = 0.03; *g* = −0.8; serenity: *F*_(1,56)_ = 7.13, *P* = 0.01; *g* = 0.8; shyness: *F*_(1,56)_ = 10.24, *P* < 0.001; *g* = −1.0; fear: *F*_(1,56)_ = 9.25, *P* < 0.001; *g* = −0.5). The pattern of* post hoc* effects was the same as that observed for hyperactivity-impulsivity symptoms on the ASRS and depression and anxiety scores (*P* values <0.05).

Regarding quality of life assessed with the AAQoL questionnaire (Table 2), there was a main effect of health status for all variables, indicating worse quality of life in the ADHD patients (life productivity: *F*_(1,56)_ = 30.45, *P* < 0.001; psychological health: *F*_(1,56)_ = 16.50, *P* < 0.001; life outlook: *F*_(1,56)_ = 21.20, *P* < 0.001; relationships: *F*_(1,56)_ = 24.64, *P* < 0.001; total quality of life: *F*_(1,56)_ = 35.17, *P* < 0.001). There were also interactions between session and intervention for all domains (life productivity: *F*_(1,56)_ = 19.06, *P* < 0.001; *g* = 1.3; psychological health: *F*_(1,56)_ = 8.67, *P* < 0.001; *g* = 0.9; life outlook: *F*_(1,56)_ = 14.73, *P* < 0.001; *g* = 1.0; relationships: *F*_(1,56)_ = 8.76, *P* < 0.001; *g* = 0.9; total quality of life *F*_(1,56)_ = 28.13, *P* < 0.001; *g* = 1.5). These effects followed the same general pattern described above, that is, no significant difference between conditions at baseline, and improvement after MAP at endpoint compared with baseline and with participants who did undergo intervention (*P* values <0.001); additionally, there was no significant difference in performance between sessions in participants who did not participate in MAP.

### 3.4. Effects of MAP on Attentional Performance Measures (Table 3)

We employed the same GLMs and factors that were used to evaluate affective measures. The pattern of effects on attention performance differed from the pattern of effects on affective ratings. On the ANT, there were no effects of health status alone and no interaction between this factor and others. Regarding the effects of session (practice effects), we obtained various indications that the task was sensitive to practice effects because performance was better at the endpoint than at baseline for the variables executive control/conflict (*F*_(1,56)_ = 12.80; *P* < 0.001), hit RT (*F*_(1,56)_ = 11.57; *P* < 0.001), accuracy (*F*_(1,56)_ = 31.33; *P* < 0.001), omission errors (*F*_(1,56)_ = 50.55; *P* < 0.001), hit RT SE (*F*_(1,56)_ = 7.29; *P* = 0.01), and hit SE block change (*F*_(1,56)_ = 4.90; *P* = 0.03).

However, there were also positive effects of the MAP intervention irrespective of health status (see Figure 2 for the effect sizes of the intervention). The intervention interacted with session for the hit RT block change measure (*F*_(1,56)_ = 8.16; *P* = 0.01; *g* = −0.9). The pattern of* post hoc* contrasts was the same as that observed for most of the affective measures: there were no significant differences between conditions at baseline, but at endpoint the MAP intervention improved scores compared with baseline and with the scores of the participants who did not participate in the intervention (*P* values <0.05), which were not statistically different at both sessions.

Regarding the CPT II (Table 3), a main effect of health status was found for commission errors (*F*_(1,56)_ = 6.88; *P* = 0.01) and detectability (*F*_(1,56)_ = 4.06; *P* = 0.05); the ADHD patients displayed worse scores, as expected. Furthermore, there was an interaction between session and intervention for both of these variables (commission errors *F*_(1,56)_ = 8.74; *P* < 0.001; *g* = −0.9; detectability *F*_(1,56)_ = 13.24; *P* < 0.001; *g* = 1.1), again with the same* post hoc* beneficial effects of MAP that were described for affective measures and hit RT block change on the ANT.

Changes in depression and anxiety scores did not correlate with performance changes in any of the attentional changes (*P* values >0.15). Low correlations were found between ADHD symptoms and attentional measures: inattention ratings correlated with commission errors (*R* = 0.38; *P* = 0.003) and detectability (*R* = −0.29; *P* = 0.02), while hyperactivity-impulsivity ratings correlated with commission errors (*R* = 0.35; *P* = 0.006) and detectability (*R* = −0.30; *P* = 0.02).

## 4. Discussion

Overall, we found that the adults with ADHD had worse affective ratings, quality of life, and attentional performance compared with controls and that MAP improved measures in all of these parameters, in accordance with our hypothesis. However, these effects did not show significant differences between controls and patients, a finding that we did not expect given the larger number and greater intensity of negative symptoms in the patients. Most of the MAP-induced effects reached large effect sizes, which attests to the clinical importance of our findings.

First, we should address a possible difference between the individuals who were willing to participate in MAP and those who were not. We found only one difference at baseline between these groups of individuals, which did not interact with health status; therefore, we believe that our quasiexperimental design, though not ideal, did not negatively impact our main findings of the beneficial effects of the MAP. Among the 21 subjective measures evaluated at baseline, the ADHD patients and healthy controls who wanted to participate in the intervention (meditation ADHD and meditation control, resp.) rated themselves similarly to those who did not want to participate (no intervention ADHD and no intervention control, resp.). These measures were related to the scores of ASRS measures of inattention, hyperactivity-impulsivity, Beck Depression, STAI anxiety, PANAS-X measures of negative affect, positive affect, fear, hostility, guilt, sadness, joviality, self-assurance, shyness, fatigue, serenity, and surprise, as well as the quality of life measures, that is, life productivity, psychological health, life outlook, relationships, and total quality of life, except that the meditation ADHD and meditation control (intervention groups) reported being less attentive compared with the no intervention ADHD and no intervention control groups in the PANAS-X attentiveness score. However, there was no objective indication of worse attentiveness among these individuals on any of the 22 objective attentional measures on the ANT and CPT II measures. Hence, we believe that this sole effect does not reflect an actual difference in the profile of the participants who did and did not undertake MAP.

Regarding subjective ratings of mood, the ADHD patients reported more ADHD symptoms, depression, and anxiety, as well as more negative and less positive affect compared with healthy controls, as expected [24, 25]. MAP improved these symptoms in the ADHD patients, in accordance with the results of Mitchell et al. [43] and Zylowska et al. [15], and in the healthy controls, as reported by Astin [9], Jha et al. [10], and van den Hurk et al. [11]. Both the participants with ADHD and the healthy control participants found the intervention rewarding, as determined by their high satisfaction ratings, and both groups were motivated by MAP based on both their attendance of the weekly sessions and the frequency and extent of their home practices. In comparison, the mood ratings of the participants who did not participate in the MAP program did not change between sessions. This indicates that the affective state was stable during the period during which the program took place. Furthermore, the experience of having completed the questionnaires previously did not alter the participants' subjective ratings, so test-retest reliability seems to have been adequate for these measures.

Regarding the assessment of quality of life with the AAQol, which focuses on ADHD problems, we also showed that the ADHD patients had worse ratings than the controls did, as is commonly found [20], corroborating our hypothesis. Additionally, the interaction between session and intervention mirrored the above-mentioned beneficial MAP-induced effect on mood; the patients and controls reported greater life productivity and psychological health, a better outlook on life, and improved relationship issues after the intervention, and all effects sizes were large. This finding also confirms findings that nonclinical populations experience improved quality of life after mindfulness training [7, 8].

Concerning performance on attentional tasks, like others, we observed that the ADHD patients showed impairment on the CPT II measures commission errors, an indication of impulsivity [38–40], and detectability, or the ability to distinguish relevant from irrelevant information [30], which is related to the concept of executive control [26]. The classic ANT measures were not impaired in the ADHD patients at baseline. This finding supports those of Lundervold et al. [25] but differs from those of Lampe et al. [36], who showed executive deficits, which we found using the CPT II. Hence, it seems ideal to use both of these tasks to evaluate executive attentional deficits in ADHD.

Concerning the attentional effects of MAP, the CPT II measures that were impaired in ADHD patients at baseline compared with controls (i.e., commission errors and detectability) were improved by the intervention. Nonetheless, these effects were not specific to ADHD patients and were also observed in the controls (interaction of intervention and session). These results indicate better MAP-induced regulation of behavior and/or self-control of impulsive tendencies [66] with the consequential potential for improving attention and emotion [6, 27]. These attentional changes, though, are most likely not wholly secondary to improvements in mood and ADHD symptoms, considering that correlations were not present or low. This confirms that MAP can alter brain functioning related to attentional performance [12, 13, 31, 32, 41, 45].

Despite repetition of the attentional tasks (baseline and endpoint), we did not find any measure on the CPT II that exhibited practice effects (main effect of session with no interaction with other factors), indicating that results were not contaminated by a lack of test-retest reliability. In contrast, various variables obtained from the ANT were susceptible to repetition, as Ishigami and Klein [67] found, including the executive/conflict measure, for which performance improved at endpoint compared with baseline. Various other measures derived from the ANT that are classical CPT II measures (see [25]) were also improved at endpoint (hit RT, hit RT SE, hit SE block change, omission errors, and accuracy) irrespective of health status. Hence, MAP's positive executive effects on the ANT in ADHD patients, as reported by Zylowska et al. [15], may have been caused by practice and not the intervention itself. Note that these authors only compared performance between baseline and after MAP in ADHD patients and did not include a nonintervention control group. In contrast and in agreement with our results, Mitchell et al. [43], who controlled for practice effects, found that the same MAP protocol that was used here and by Zylowska et al. [15] had no beneficial effect on the classic ANT measures. Mitchell et al. [43] also failed to find MAP-induced effects on the CPT II, in contrast with our findings; this difference may be related to a lack of power, as their sample of ADHD patients was smaller.

Despite these practice effects, we did show objective beneficial changes resulting from MAP on a variable that was not evaluated in the latter studies. The measure hit RT block change, which was derived from the ANT data, improved after the MAP intervention, and this can be attributed to increases in the ability to sustain attention or vigilance [31, 32], an ability that is impaired in adults with ADHD [68] and seems to improve after mindfulness practices [5]. However, this effect is not commonly found when the CPT II is used [38]. Interestingly, this effect was not shown for the analogous measure obtained from the CPT data, or for the alerting variable of the ANT, a concept that is highly similar to sustained attention/vigilance (see [43]). Hence, it seems to be useful to calculate CPT measures using the ANT results, as proposed by Lundervold et al. [25], because doing so increases the likelihood of detecting susceptibility to practice effects and changes in attentional performance.

There are indications that mindfulness practices can improve executive attention in inexperienced meditators, especially after short-term programs (see [5]), but we did not find such improvements using the ANT. However, we did show a MAP-induced improvement in the CPT II variables detectability and commission errors, in contrast with some studies that used this task (see [5]). These measures are related to the concept of executive attention because they involve discriminating relevant from irrelevant visual signals, as well as inhibitory processes [26]. According to Fernandez-Duque et al. [26], this type of executive functioning relates to better metacognitive monitoring, which involves control processes such as conflict resolution and emotional regulation. This fits nicely with the improvement in affect found here.

Thus, a series of our findings indicated that the ANT and CPT are complementary in the present setting and should be used together when evaluating MAP and/or ADHD attentional effects. The measures were differently sensitive to practice effects; CPT measures derived from ANT data indicated MAP-induced improvement in sustained attention that the CPT did not, and the variables on the CPT that indicate executive functioning were positively affected by MAP, while those variables on the ANT were not. One possible reason for this is that these tasks have different characteristics. One main difference is that, in the ANT paradigm [33], the participant must respond to all trials; therefore, impulsivity, which is one of the main symptoms of ADHD [17–19], cannot be shown. In other words, commission errors and detectability cannot be determined in this task, and these variables are susceptible to ADHD and were sensitive to improvement with MAP. Another aspect of the ANT is that it involves a fixed time interval of 400 ms between the cues and the target. This increases the predictability of the need to respond, which is unlike the CPT, in which interstimulus intervals vary. This is important because it has been shown that adults with ADHD have deficits related to the estimation of time intervals [69], which may contribute to the usefulness of the CPT for detecting their attentional problems [37], as we found here. On the other hand, this lack of variability in the interstimulus intervals of the ANT may have enabled MAP-induced sustained attention improvement to be detected.

One possible hypothesis for the comparable improvement in mood, quality of life, and attentional performance between the ADHD patients and healthy controls after MAP is that our control group was small. With a larger sample, differences might have become apparent. Additionally, these similar results between groups may have resulted from the use of a treatment program that was developed specifically for adults with ADHD (see details in Zylowska et al. [15] and Zylowska [59]). Thus, the intervention used in our study may have led to specific improvement in aspects that are impaired in this clinical condition. It is therefore possible that other mindfulness programs may lead to different attentional performance improvements in healthy adults, as found by Tang et al. [70]. This is especially true considering that the effects found here for the control group indicated that attention has room for improvement by MAP, even in healthy individuals.

There were limitations to our study apart from the small number of control participants. Like Zylowska et al.'s [59] study, our study was not a randomized trial, as would have been ideal, because we were not able to recruit a sufficiently large sample of subjects who fit the eligibility criteria and were willing to practice meditation. Likewise, in our study, the experimenter was not blind to the treatment, as seems to have occurred in Zylowska et al.'s work. However, we believe that this did not compromise our data because the ADHD patients and controls who agreed to participate in the intervention and those who did not did not differ in terms of demographic variables or IQ or on any subjective measure except inattention.

It can also not be excluded that the awareness of participants that they would be submitted to the intervention biased the observed effects. However, we would expect this to influence only subjective measures and not the attentional ones, which were also improved, suggesting that our data do not reflect pure expectation effects. The possibility that patients with different ADHD subtypes would have reacted differently to the MAP intervention cannot be excluded, especially because people with different subtypes seem to perform differently on the ANT [71] and CPT II [72]. It must be considered, however, that new guidelines do not propose ADHD subtypes as separate clinical representations because they are not developmentally stable (see [20]). Age and gender specific effects must also be investigated, as should the impact of MAP on nonmedicated and medicated patients. Unfortunately, our sample was not large enough to conduct the latter types of analyses. Finally, our indirect measures of MAP-induced improvement in brain functioning in the form of better attentional performance should incentivize future investigations into the cognitive systems that are at play in this phenomenon.

### 4.1. Conclusions

Mindfulness awareness practices improved affective symptoms, quality of life, and attentional performance (sustained attention and executive control) in adult ADHD patients and controls. Hence, this intervention can be considered a useful complementary treatment for adults with ADHD that also has the potential to enhance attention, mood, and quality of life in nonclinical populations.

## Figures and Tables

**Table 1:** Mean (standard deviation) of demographic information per group (control and patients with attention deficit hyperactivity disorder (ADHD) submitted to mindful awareness practices (MAP) or to no intervention) and statistical comparison between them, times of at-home practice, and rating of satisfaction with the programme in the groups submitted to the MAP.

Variable	MAP control (*N* = 8)	No intervention control (*N* = 9)	MAP ADHD (*N* = 21)	No intervention ADHD (*N* = 22)	*P*
Demographics					
Gender (men/women)	3/5	4/5	11/10	12/10	
Age (years)	26.9 (3.9)	28.7 (5.5)	31.2 (7.5)	31.7 (7.8)	0.32
Education (years)	16.0 (2.8)	15.9 (2.0)	14.6 (2.4)	15.1 (3.2)	0.53
IQ Raven (number correct)	54.4 (1.7)	52.2 (7.4)	52.6 (3.7)	49.4 (7.4)	0.16
At-home practices (min)^*^					
Week 1	15.6 (11.2)		32.9 (17.6)		
Week 2	21.3 (7.9)		19.8 (14.1)		
Week 3	31.3 (18.1)		48.1 (27.3)		
Week 4	37.5 (10.4)		52.9 (48.8)		
Week 5	45.0 (10.7)		50.5 (42.8)		
Week 6	54.4 (15.9)		49.7 (50.7)		
Week 7	45.0 (16.0)		42.9 (57.6)		
Week 8	48.8 (10.6)		33.6 (20.6)		
Satisfaction (1–10 score)	9.3 (0.9)		9.3 (0.9)		0.82

^*∗*^No effect of health status or interaction with week, but there was an effect of week [*F* (7, 189) = 4.35; *P* < 0.001]: practice in weeks 3–8 > weeks 1-2 (*P* < 0.001).

**Table 2:** Mean (standard deviation) scores on the Adult ADHD Self-Report Scale (ASRS), Beck Depression Inventory (BDI), State-Trait Anxiety Inventory (STAI-T), Positive and Negative Affect Schedule—expanded form (PANAS-X), and Adult ADHD Quality of Life Questionnaire (AAQoL) per group (control and patients with attention deficit hyperactivity disorder (ADHD) submitted to mindful awareness practices (MAP) or to no intervention) at baseline and after the intervention period (endpoint) and significant effects.

Measure	Meditation		No intervention		Meditation		No intervention		
	Control		Control		ADHD		ADHD		Significant
	(*N* = 8)		(*N* = 9)		(*N* = 21)		(*N* = 22)		effects
	Baseline	Endpoint	Baseline	Endpoint	Baseline	Endpoint	Baseline	Endpoint	
ASRS									
Inattention	15.5 (3.3)	11.9 (3.4)	10.0 (2.8)	11.1 (5.1)	29.4 (3.9)	21.9 (5.1)	27.4 (5.9)	26.2 (4.9)	H; S × I
Hyperactivity-impulsivity	14.6 (4.1)	11.3 (3.5)	10.8 (4.4)	11.9 (3.2)	21.9 (7.1)	17.9 (5.6)	23.1 (6.9)	21.9 (6.5)	H; S × I
Depression (BDI)	10.0 (8.2)	4.6 (2.7)	4.0 (3.0)	4.1 (2.0)	14.0 (7.9)	10.6 (9.5)	12.4 (7.7)	12.6 (8.2)	H; S × I
Anxiety (STAI-T)	42.9 (6.2)	37.1 (3.3)	35.4 (3.9)	34.9 (8.5)	53.7 (11.7)	45.5 (11.3)	52.2 (11.2)	51.6 (10.2)	H; S × I
PANAS-X									
Negative affect	2.1 (1.1)	1.4 (0.2)	1.5 (0.4)	1.5 (0.4)	2.2 (0.8)	1.8 (0.7)	2.3 (0.7)	2.4 (0.8)	H; S × I
Positive affect	2.5 (0.8)	3.2 (0.9)	3.0 (0.5)	3.0 (0.5)	2.3 (0.6)	2.6 (0.7)	2.7 (0.8)	2.4 (0.7)	H; S × I
Fear	2.0 (1.0)	1.3 (0.2)	1.4 (0.2)	1.7 (0.6)	1.9 (0.7)	1.6 (0.7)	2.0 (0.6)	2.0 (0.6)	S × I
Hostility	1.9 (0.8)	2.6 (3.8)	1.4 (0.4)	1.4 (0.2)	2.1 (0.9)	1.7 (0.7)	2.1 (0.8)	2.1 (0.7)	
Guilt	2.0 (0.9)	2.6 (3.8)	1.5 (0.4)	1.7 (0.7)	2.0 (0.9)	1.5 (0.5)	2.0 (0.9)	2.2 (1.0)	
Sadness	2.0 (0.7)	1.3 (0.3)	1.4 (0.4)	1.6 (0.8)	2.2 (1.1)	1.8 (1.1)	2.1 (1.0)	2.4 (2.1)	H; S × I
Joviality	2.7 (0.9)	3.3 (1.1)	3.0 (0.6)	2.7 (0.5)	2.2 (0.6)	2.5 (0.9)	2.5 (0.8)	2.2 (0.7)	H; S × I
Self-assurance	2.1 (0.6)	2.7 (0.9)	3.3 (1.2)	3.2 (1.2)	2.0 (0.5)	2.7 (1.0)	2.5 (0.9)	2.4 (0.7)	H; S × I
Attentiveness	3.1 (0.8)	3.3 (0.7)	3.3 (0.7)	3.4 (0.5)	2.2 (0.7)	2.8 (0.7)	2.5 (0.9)	2.4 (0.8)	H; S × I
Shyness	2.2 (1.0)	1.4 (0.4)	1.9 (0.6)	1.7 (0.4)	2.0 (0.8)	1.3 (0.4)	2.1 (0.8)	2.0 (0.8)	S × I
Fatigue	2.6 (1.3)	2.0 (0.5)	2.1 (0.8)	2.0 (0.7)	2.9 (0.7)	2.3 (1.0)	2.5 (0.7)	2.6 (0.9)	H; S × I
Serenity	2.7 (0.6)	3.2 (0.6)	2.9 (0.6)	3.0 (0.7)	2.2 (0.6)	2.7 (0.7)	2.4 (0.7)	2.3 (0.5)	H; S × I
Surprise	1.6 (0.7)	1.6 (0.7)	1.7 (0.8)	1.5 (0.5)	1.5 (0.6)	1.7 (0.8)	1.9 (0.9)	1.7 (0.6)	
AAQoL									
Life productivity	64.5 (15.5)	77.8 (10.2)	63.4 (21.2)	61.3 (19.6)	34.4 (13.2)	57.1 (10.4)	38.4 (20.4)	43.3 (17.0)	H; S × I
Psychological health	60.9 (16.4)	65.3 (13.1)	64.4 (17.2)	58.3 (17.9)	39.5 (15.1)	57.7 (17.5)	39.4 (19.1)	42.2 (16.2)	H; S × I
Life outlook	56.3 (12.2)	76.4 (13.0)	66.0 (9.7)	67.5 (10.2)	48.2 (16.1)	60.4 (16.4)	45.9 (16.9)	43.2 (15.5)	H; S × I
Relationships	63.1 (20.2)	71.3 (14.3)	70.0 (16.4)	65.6 (18.1)	43.1 (15.9)	60.7 (13.2)	43.4 (18.9)	44.1 (15.0)	H; S × I
Total quality of life	61.2 (12.5)	72.7 (9.3)	66.0 (14.3)	63.2 (12.5)	41.3 (11.8)	59.0 (12.1)	41.8 (14.1)	43.2 (11.9)	H; S × I

H: effect of health status (ADHD versus controls); S: effect of session (baseline versus endpoint) or practice effect; I: effect of intervention (MAP versus no intervention); ×: interaction of factors (*P* values < 0.05). See text for details on the statistical analysis.

**Table 3:** Mean (standard deviation) scores on the Attentional Network Task (ANT) and the Conners Continuous Performance Test (CPT II) per group (control and patients with attention deficit hyperactivity disorder (ADHD) submitted to mindful awareness practices (MAP) or to no intervention) at baseline and after the intervention period (endpoint) and significant effects.

Measure	Meditation		No intervention		Meditation		No intervention		
	Control		Control		ADHD		ADHD		Significant
	(*N* = 8)		(*N* = 9)		(*N* = 21)		(*N* = 22)		effects
	Baseline	Endpoint	Baseline	Endpoint	Baseline	Endpoint	Baseline	Endpoint	
ANT									
Alerting	33.6 (22.2)	43.8 (28.8)	33.4 (21.4)	35.8 (27.0)	32.0 (29.0)	39.3 (19.7)	37.4 (28.8)	46.0 (27.1)	
Orientation	29.3 (11.6)	30.1 (13.8)	16.8 (14.4)	26.4 (22.7)	29.8 (30.5)	32.1 (21.2)	32.2 (41.0)	26.6 (23.8)	
Executive control/conflict	114.8 (38.9)	88.1 (38.0)	198.2 (68.2)	114.3 (55.2)	184.6 (98.5)	140.1 (77.2)	150.7 (64.9)	107.5 (69.9)	S
Hit RT (ms)	535.1 (75.5)	488.8 (46.8)	612.2 (114.6)	597.1 (142.1)	577.4 (123.0)	537.4 (90.2)	546.4 (101.8)	511.9 (89.8)	S
Accuracy (number)	274.9 (9.6)	284.0 (4.3)	274.8 (8.1)	278.1 (9.0)	272.0 (11.4)	278.5 (9.3)	273.4 (9.9)	277.0 (9.1)	S
Omissions (number)	5.4 (3.3)	3.0 (3.0)	5.3 (3.4)	3.8 (3.1)	7.2 (4.5)	4.4 (3.5)	7.0 (3.4)	4.6 (3.3)	S
Hit RT SE	16.1 (6.2)	13.2 (7.7)	18.6 (4.9)	15.9 (17.3)	16.4 (14.0)	11.3 (7.4)	20.2 (11.3)	11.9 (6.6)	S
Variability of SE	1.3 (1.0)	1.4 (1.6)	1.9 (1.1)	2.1 (1.5)	2.1 (1.6)	1.6 (1.6)	2.3 (1.8)	1.6 (1.1)	
Hit RT block change	−17.8 (17.4)	−26.1 (13.4)	−18.5 (25.1)	−10.1 (31.1)	−5.5 (22.9)	−19.7 (16.0)	−12.4 (22.0)	−1.9 (15.6)	S × I
Hit SE block change	−0.18 (0.66)	−1.18 (1.50)	−1.01 (1.74)	−0.99 (2.12)	−0.18 (2.36)	−1.29 (1.54)	0.08 (1.60)	−0.93 (2.18)	S
CPT II									
Omission errors (number)	1.4 (2.0)	0.0 (0.0)	1.7 (2.7)	1.1 (1.3)	2.4 (4.3)	2.1 (3.2)	3.8 (7.6)	2.8 (5.7)	
Commission errors (number)	9.1 (4.0)	4.5 (3.6)	7.2 (4.4)	7.7 (4.4)	15.1 (7.8)	10.6 (8.4)	12.5 (9.8)	12.9 (9.7)	H; S × I
Hit RT (ms)	385.8 (76.0)	407.0 (55.0)	408.5 (68.5)	396.9 (64.3)	386.3 (75.0)	397.1 (69.3)	376.9 (115.6)	378.2 (67.8)	
Variability of SE	5.9 (1.5)	5.1 (2.3)	7.6 (1.9)	7.8 (4.7)	7.9 (3.6)	8.4 (8.4)	9.7 (8.8)	9.8 (12.5)	
Hit RT SE	5.2 (1.6)	5.0 (1.6)	5.5 (1.4)	5.4 (2.0)	6.0 (2.2)	5.8 (3.0)	6.5 (2.6)	6.2 (3.7)	
Detectability (*d*′)	0.9 (1.3)	1.3 ( 0.4)	1.1 (0.4)	1.0 (0.3)	0.7 (0.4)	0.9 (0.5)	0.9 (0.7)	0.8 (0.6)	H; S × I
Hit RT block change	−0.014 (0.035)	−0.013 (0.028)	−0.003 (0.021)	−0.003 (0.017)	0.001 (0.034)	−0.004 (0.025)	−0.011 (0.030)	−0.003 (0.027)	
Hit SE block change	−0.030 (0.059)	0.033 (0.058)	0.021 (0.065)	0.004 (0.081)	0.001 (0.063)	0.016 (0.060)	0.002 (0.103)	0.001 (0.066)	
Hit RT ISI change	0.058 (0.014)	0.061 (0.011)	0.046 (0.039)	0.052 (0.040)	0.046 (0.043)	0.050 (0.043)	0.066 (0.030)	0.058 (0.035)	
Hit SE ISI change	−0.015 (0.120)	0.019 (0.077)	−0.019 (0.100)	−0.006 (0.112)	−0.030 (0.099)	−0.017 (0.133)	0.002 (0.104)	0.027 (0.117)	
Response style (*β*)	0.6 (0.8)	0.7 (0.6)	0.9 (0.8)	1.7 (1.5)	1.1 (2.4)	0.7 (0.6)	1.0 (1.1)	0.5 (0.4)	
Perseverations	0.0 (0.0)	0.0 (0.0)	0.4 (0.7)	0.4 (0.7)	0.7 (1.5)	2.4 (5.9)	1.8 (3.6)	1.7 (3.4)	

Hit: correct responses; RT: reaction time; SE: standard error of the mean; ISI: interstimulus interval; H: effect of health status (ADHD versus controls); S: effect of session (baseline versus endpoint) or practice effect; I: effect of intervention (MAP versus no intervention); ×: interaction of factors (*P* values < 0.05). See text for details on the statistical analysis.
